# Non-convulsive Status Epilepticus in *SEMA6B*-Related Progressive Myoclonic Epilepsy: A Case Report With Literature Review

**DOI:** 10.3389/fped.2022.859183

**Published:** 2022-04-28

**Authors:** Jing Duan, Yan Chen, Zhanqi Hu, Yuanzhen Ye, Tian Zhang, Cong Li, Qi Zeng, Xia Zhao, Jiahui Mai, Yang Sun, Chao Liu, Wenxin Zheng, Yuhan Xiao, Jianxiang Liao, Li Chen

**Affiliations:** ^1^Department of Neurology, Shenzhen Children’s Hospital, Shenzhen, China; ^2^Department of Epilepsy Surgery, Shenzhen Children’s Hospital, Shenzhen, China; ^3^Department of Bioinformatics, Berry Genomics Co., Ltd., Beijing, China

**Keywords:** non-convulsive status epilepticus (NCSE), *SEMA6B*, progressive myoclonic epilepsy, piracetam, frameshift mutation

## Abstract

Progressive myoclonic epilepsy (PME) is a group of rare diseases characterized by progressive myoclonus, cognitive impairment, ataxia, and other neurologic deficits. PME has high genetic heterogeneity, and more than 40 genes are reportedly associated with this disorder. *SEMA6B* encodes a member of the semaphorin family and was first reported to cause PME in 2020. Herein, we present a rare case of PME due to a novel *SEMA6B* gene mutation in a 6-year-old boy born to healthy non-consanguineous Chinese parents. His developmental milestones were delayed, and he developed recurrent atonic seizures and myoclonic seizures without fever at 3 years and 11 months of age. He experienced recurrent myoclonic seizures, non-convulsive status epilepticus (NCSE), atonic seizures, and atypical absence seizures during the last 2 years. At different time points since onset, valproic acid, levetiracetam, piracetam, and clobazam were used to control the intractable seizures. Notably, NCSE was controlled by a combination of piracetam with clobazam and valproic acid instead of intravenous infusion of midazolam and phenobarbital. Due to the limited number of cases reported to date, the clinical description of our case provides a better understanding of the genotype–phenotype correlations associated with PME and indicate that piracetam may be effective against NCSE in patients with *SEMA6B*-related PME.

## Introduction

Progressive myoclonic epilepsy (PME) is a group of disorders associated with focal and generalized seizures, myoclonus, and progressive neurological deficits (1). PME primarily presents as myoclonic seizures; however, generalized tonic–clonic, tonic, and atypical absence seizures have also been reported (2). PME is a genetically heterogeneous disorder, and the common causative genes associated with PME are *CTSB*, *NHLRC1*, *EPM2A*, *MTTK*, *CLN2*, and *CLN6*;

besides these, there are more than 30 other causative genes which are relatively rarely associated with PME (3–6).

*SEMA6B* (OMIM:608873) was first reported to be associated with PME in 2020 by Hamanaka (7), and only 11 patients with PME have been reported to date. The small number of mutations observed in *SEMA6B* in patients with PME has limited our understanding of how *SEMA6B* plays a role in the pathogenesis of PME. In addition, the treatment experience is also very limited (8).

In this report, we present the clinical and genetic data of a child with PME caused by a *SEMA6B* gene variant. The new genetic evidence presented herein strengthens the gene–disease relationship, and we believe this case report will improve clinicians’ understanding of the disease. In addition, this report expands the mutation spectrum of *SEMA6B* to provide early diagnosis and genetic counseling to such patients. Furthermore, to our knowledge, this is the first report to mention piracetam as an option to manage non-convulsive status epilepticus (NCSE) in PME.

## Case Presentation

The proband was a 6-year-old boy born to healthy non-consanguineous Chinese parents. There was no remarkable medical history in the family. He had a healthy younger sister and was born at full term following an uneventful pregnancy.

His developmental milestones were delayed, and he did not have head control until the age of 10 months, could not sit without support until 17 months, and could walk without support only at 32 months, but with an unsteady gait. Hence, he was diagnosed with developmental delay at a local hospital. At 3 years and 11 months, he developed recurrent myoclonic seizures without fever. His parents reported his seizure onset as head nodding or fall. The video EEG monitoring showed delta waves during wakefulness, and interictal EEG was characterized by multifocal spikes or spike-and-wave discharges, sharp waves, slow waves, and generalized spike or polyspike–wave complexes. Ictal EEG showed bursts of 1.5–2.5 Hz generalized spike-wave complex while surface electromyogram showed a brief silent period (<200 ms), symptoms included nodding or drop attacks, consistent with negative myoclonus. Surface electromyography also captured hypersynchronous myoclonus electromyography discharges (<200 ms) while myoclonic jerks involving the trunk and limbs, consistent with myoclonus. He was given valproic acid (VPA; max dosage = 28.6 mg/kg.d) and levetiracetam (LEV; max dosage = 66.7 mg/kg.d), and he remained seizure-free for more than 1 year. Because of the motor regression caused by this bout of seizures, he could not walk at all until becoming seizure-free at 4 years of age. During the year when he was seizure-free, he could walk with a steady gait and learned more skills. At 5 years of age, he experienced recurrent myoclonic seizures with head nodding. He was administered clonazepam (CZP; max dosage = 0.67 mg/kg.d), topiramate (TPM; max dosage = 1.7 mg/kg.d), zonisamide (ZNS; max dosage = 12.9 mg/kg.d), and ketogenic diet, but none of these were effective. At 5.5 years of age, he was admitted to our hospital for the first time, and his interictal electroencephalogram (EEG) showed bursts of diffuse irregular spike-and-wave discharges, whereas his ictal EEG showed a burst of generalized spike-and-wave discharges; furthermore, his video monitoring revealed myoclonic seizures and atypical absence seizures. Clobazam (CLB; max dosage = 0.94 mg/kg.d) was added, and this kept him seizure-free for 3 months. Perampanel (PER; max dosage = 0.12 mg/kg.d) was added after the seizures returned, and he was seizure-free for another 3 months.

**FIGURE 1:**
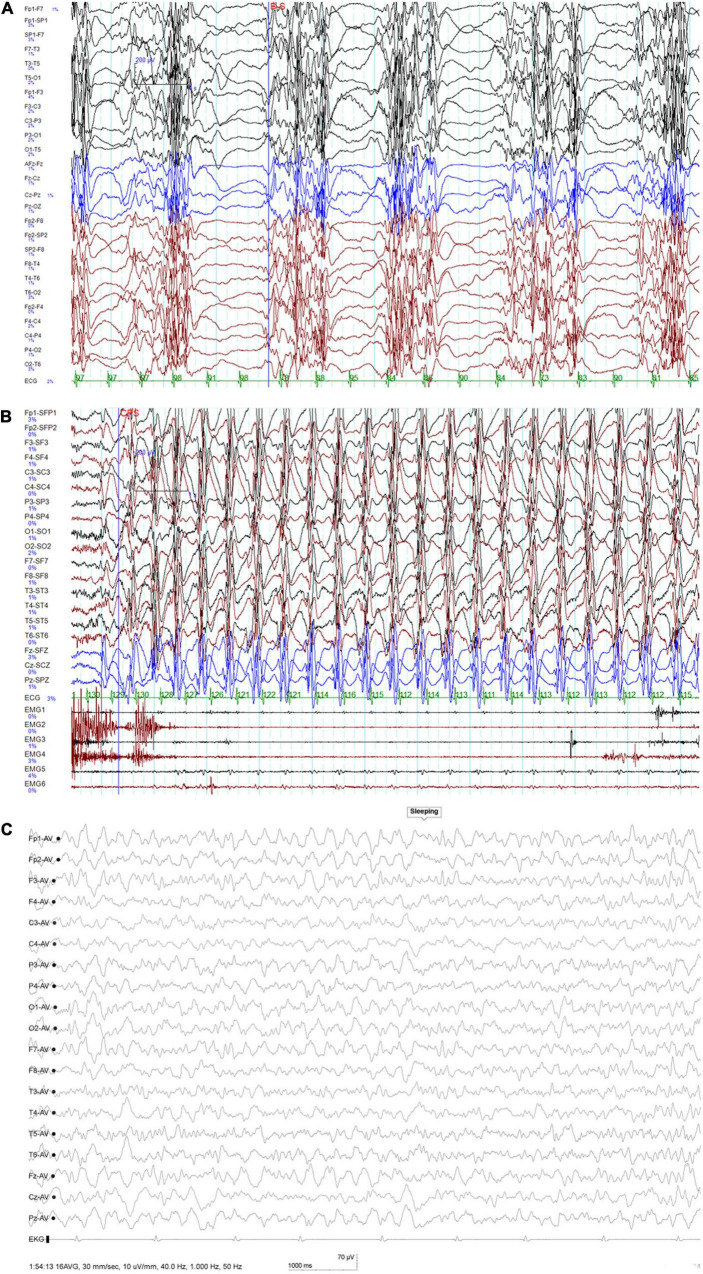
**(A)** Interictal EEG at 6 years of age showing periodic occurrence of diffuse paroxysmal multiple spike-and-slow-wave complexes and usually a second or so of suppression seen thereafter. **(B)** Ictal EEG at 6 years of age indicating slow waves of high amplitude at 2–3 Hz with spike-and-wave complexes. **(C)** At 6.5 years of age, this EEG was taken when the seizure was controlled: no ictal episode was noted during monitoring.

At 6 years of age, the recurrent seizures made him lethargic all the time, and he suffered from dysphagia with constant drooling. Long-term video EEG was performed as soon as he visited our center. The video EEG showed continuous diffuse delta activity during wakefulness as the markedly abnormal background; interictal EEG showed periodic diffuse paroxysmal multiple spike-and-wave complex, and usually, a second or so of suppression was seen afterward. Meanwhile, ictal EEG showed continuous discharges of 2–2.5 Hz generalized slow spike-and-wave complexes and polyspike-and-wave complexes, the video capture revealed the symptoms of continuous staring, subtle behavioral arrest, impaired awareness, constant blinking, and myoclonus of the limbs and eyelids. The ictal status could last for hours, which was regarded as NSCE according to its diagnosis criterion. His hypotonia became very severe, and he showed little response to language and little ability to verbally communicate. He was admitted to the intensive care unit for NCSE, pneumonia, and lethargy. NCSE persisted despite high-dose midazolam (dosage, 20 μg/kg⋅min IV drip) and an IV drip of phenobarbital (40 mg/kg.d), and mechanical ventilation was required for 6 days at a time in the intensive care unit. During this period, interictal EEG during sleep showed periodic occurrence of diffuse paroxysmal multiple spike-and-slow-wave complexes, and usually, a second or so of suppression was seen thereafter (Figure 1A). Meanwhile, ictal EEG indicated diffuse slow waves of high amplitude at 2–3 Hz with spike-and-wave complexes (Figure 1B) along with impaired awareness and subtle clinical ictal phenomena. He was successfully weaned from the mechanical ventilator after withdrawal of phenobarbital and midazolam. After administering high-dose oral piracetam (max dosage, 225 mg/kg.d) combined with oral clobazam (CLB; dosage, 0.68 mg/kg.d) and valproic acid (VPA; dosage, 21.6 mg/kg.d), he regained consciousness and the seizures gradually decreased; the seizures completely stopped in 10 days. The EEG taken after seizure remission showed no ictal episode. After 40 days, both his motor skills and language skills had returned to baseline levels. At the last follow-up, which was 4 months later, he was still seizure free.

Repeated cranial MRIs were unremarkable. Extensive laboratory testing, including liver and renal function, organic acids in urine, and amino acids in plasma, showed normal results. During his last follow-up at 6.5 years of age, he had been seizure-free for 4 months with the combination treatment of piracetam, clobazam, valproic acid, and zonisamide. His height, weight, and head circumference were 108.5 cm (–2 *SD*), 16.9 kg (–2 *SD*), and 48.5 cm (–2.0 *SD*). He could walk without support in an unsteady way, could respond to simple spoken requests, and could say simple words like “mama” and “dada.” His muscle tone improved as well. The EEG showed no ictal episode during monitoring (Figure 1C).

**FIGURE 2:**
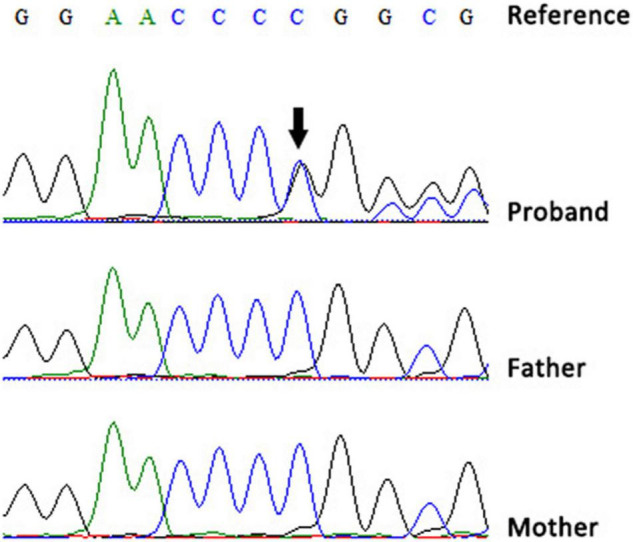
Sequencing chromatograms of the *de novo* variant in our patient (black arrow).

Blood samples were obtained from all family members for genetic analysis after obtaining written informed consent from the parents. Notably, karyotype analysis, chromosomal microarray analysis, whole-exome sequencing, and mitochondrial genetic testing performed when he was 3 years old could not identify the cause of his illness. This is because these tests were performed in 2019, which is before the first report of the *SEMA6B* gene being associated with the disease. However, at 6 years of age, trio whole-exome sequencing was performed using xGen Exome Research Panel v1.0 (IDT) on an Illumina NovaSeq 6000 (Illumina). The bioinformatics analysis was performed following the previously described pipeline (9). A *de novo* nonsense mutation (NM_032108.3:c.2023delG; p.Val675Phefs*10) in *SEMA6B* was identified, and the variant was classified as likely pathogenic according to the American College of Medical Genetics and Genomics guidelines (10). The mutation was confirmed by Sanger sequencing on an ABI 3730XL DNA Sequencer (Applied Biosystems, Thermo Fisher Scientific, United States) (Figure 2).

## Discussion

**FIGURE 3:**
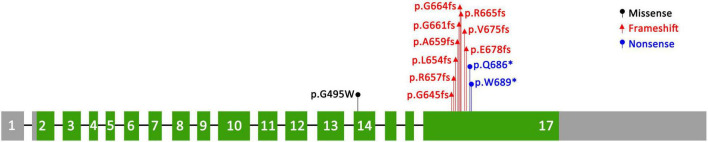
Distribution of known variants in *SEMA6B.*

We identified a novel *SEMA6B* variant in one PME patient, and this reinforces the notion that *SEMA6B* is an underlying pathogenic factor in PME. *SEMA6B* is highly expressed in the human brain and is expressed at lower levels in various other tissues (11). It encodes a protein of the class 6 semaphorin family, the members of which are involved in various processes, such as neural crest cell migration, axon guidance, and cerebellar development (12). The gene was first reported to be associated with PME by Hamanaka in 2020. They identified four frameshift variants (c.1976_1982del/p.Ala659fs, c.1991del/p.Gly664fs, c.1950_1969dup/p.Arg657fs, and c.1982_1991del/p.Gly661fs) in the last exon of *SEMA6B* in five unrelated families, all of which had a child presenting with PME. The RNA analysis of lymphoblastoid cells of an affected individual in their study showed that the mutant allele escaped nonsense-mediated mRNA decay (NMD), and they found that zebrafish expressing truncated variants of the NMD (–) region showed defective neuron development in the brain and enhanced pentylenetetrazol-induced seizure behavior. The author suggested that the gene was dominant-negative or had gain-of-function effects rather than showing haploid deficiency. Upon searching for reported cases of *SEMA6B* variants, we found seven other variants reported in the literature (6–8, 13–15). All of the variants are summarized in Table 1 and Figure 3.

**TABLE 1:** Clinical data and variants in *SEMA6B*-related progressive myoclonic epilepsy reported to date.

Clinical data	Our case	Individual 1 Hamanaka et al. (7)	Individual 2 Hamanaka et al. (7)	Individual 3 Hamanaka et al. (7)	Individual 4 Hamanaka et al. (7)	Case Li et al. (13)	PME25 Courage et al. (6)	PME83 Courage et al. (6)	Patient 1 Song et al. (14)	Patient 23^#^ Song et al. (14)	Case Li Shu et al. (15)	Case Rebecca Herzog et al. (8)
Nationality	Chinese	Japanese	Japanese	Israeli	Malaysian	Chinese	Canada	Australian	Chinese	Chinese	Chinese	NA
Gender	Male	Male	Female	Male	Female	Female	Female	Male	Female	Male	Male	Female
variant	c.2023delG (p.V675fs)	c.1950_1969dup (p.R657fs)	c.1976_1982del (p.A659fs)	c.1991del (p.G664fs)	c.1991del (p.G664fs)	c.1960_1978del (p.L654fs)	c.2032delG (p.E678fs)	c.1993delC (p.R665fs)	c.2056C>T (p.Q686*)	c.1483G C>T (p.G495W)	c.1934delG (p.G645fs)	c.2067G>A (p. W689*)
Inheritance	*De novo*	*De novo*	*De novo*	*De novo*	*De novo*	*De novo*	NA	*De novo*	*De novo*	*De novo*	*De novo*	NA
DD	+	+	+	+	+	+	+	+	+	−	+	+
Intellectual disability	Severe ID	Severe (IQ = 25 at 17 years)	Severe (IQ = 25 at 12 years)	Severe	Severe	+	Moderate ID	Severe ID	NA	NA	NA	+
Language	Few words	Few words	Few words	No words	No words	Few words	NA	NA	No words	NA	No words	Simple words
Microcephaly	+ 2.0 SD	−	−2.0 SD	−2.5 SD	+	NA	−	−	NA	NA	NA	NA
Regression	Motor and verbal skills	Motor skill and dysarthria	Motor skill	Motor and verbal skills	+	+	No definitive cognitive decline	+	+	−	NA	+
Ataxia	+	+	+	+	+	+	+	+	NA	NA	NA	+
Intention tremor	+	+	+	+	+	+	+	+	NA	NA	NA	NA
Myoclonus	+	+	+	NA	+	+	+	+	NA	NA	NA	+
Spasticity	+	+	+	NA	NA	NA	NA	NA	NA	NA	NA	+
Motor disturbance	Wheelchair	Wheelchair	Wheelchair	Wheelchair	Walking with support	Running and jumping with a little difficulty	Wheelchair	Wheelchair	Walking with support	NA	NA	Wheelchair
Brain MRI	Normal	Normal	Mild cerebellar atrophy	Small vermis	Normal	Herniation of the cerebellar tonsils	NA	NA	Normal	Normal	Normal	Normal

*^#^The pathogenicity of the variant is dubious. MRI, magnetic resonance imaging; NA, not available; Y, year.*

The symbol * stands for a stop codon.

All variants reported to date are truncation variants in the last exon of the gene, except for the c.1483G>T variant (16). The only missense mutation c.1483G>T, which was reported by Xiaozhen et al. (16), was identified *de novo* in a 3-year-old boy who had apparently normal developmental milestones. This child was referred to the hospital at the age of 2 years because of the onset of seizures with eye-rolling, cyanotic lips, consciousness lapses, and right upper limb jitter as well as a gelastic seizure lasting > 15 s. Furthermore, he had good response to combination treatment of levetiracetam and sodium valproate with no recurrence noted. The functional assay showed no changes in protein length and expression and no difference in cellular distribution; however, co-immunoprecipitation studies revealed that the variant influenced protein binding of *SEMA6B* and PlxnA2 to varying degrees. Therefore, we believe that the pathogenicity of c.1483G>T is dubious, and more evidence is needed to support it.

Clinical presentations of all 12 reported patients (including our case) are summarized in Tables 1, 2.

**TABLE 2:** Characteristics of epilepsy reported in *SEMA6B*-related progressive myoclonic epilepsy.

Clinical data	Age of seizure onset	Seizure types	Response to ASD	EEG
Our case	3 years	Focal seizures, atonic seizures, atypical absence seizures, NCSE	Intractable	Delta waves were noted during wakefulness; interictal EEG was characterized by multifocal spikes or spike-and-wave discharges, sharp waves, slow waves, and generalized spike or polyspike-and-wave complexes. Ictal EEG showed myoclonic seizures involving the trunk and limbs (4 years); diffuse slow waves were the main background during wakefulness, whereas ictal EEG showed continuous rhythmic generalized polyspike-and-wave complexes (6 years).
Individual 1 Hamanaka et al. (7)	6 years	GTCS, absence seizures, atonic seizure	Intractable	Abnormal discharge in the right hemisphere (6 years), burst of diffuse irregular spikes and slow waves (9 years), and diffuse spike and slow waves in the frontal, parietal and temporal regions (14 years)
Individual 2 Hamanaka et al. (7)	11 months	GTCS; complex partial seizures; atonic seizures	Intractable	Diffuse slow waves (2–3 Hz) and spike-and-wave discharges in the bilateral frontal region (3 years and 4 years), diffuse theta waves (4–5 Hz) and spike-and-wave bursts (2–3 Hz; 9 years), multifocal spikes in left parietal region and bilateral frontal regions (12 years), multispikes in the left occipital region (13 years), and slow waves at baseline (23 years)
Individual 3 Hamanaka et al. (7)	2 years	Absence seizures	Responsive	Abnormal background activity (1 year), slow abnormal sleep features with a paucity of sleep spindles (13 years)
Individual 4 Hamanaka et al. (7)	4 years	Atonic seizures	Intractable	Focal bifrontal epileptiform discharges accentuated during sleep (4 years), frequent frontocentral discharges during the awake state (5 years), and frequent intermittent slow spikes in the right posterior region (11 years)
Case Li et al. (13)	4 years	Atonic seizures	Responsive	Diffuse spike-and-wave bursts (1.5–2.5 Hz), particularly in the frontal regions, temporary increase in myoelectric activity, interictal widespread spina wave (1.5–2.0 Hz) released continuously and irregularly (6 years)
PME25 Courage et al. (6)	NA	Tonic–clonic seizure, possible absence and focal seizures	NA	NA
PME83 Courage et al. (6)	NA	Drop attacks and absence seizures	NA	NA
Patient 1 Song et al. (14)	2 years	Atonic seizures, complex partial seizures, atypical absence seizures	Intractable	Generalized slow spike-and-wave discharges (1.6–2 Hz), demonstrating intermittent discharges during sleep and continuous spike-waves during sleep
Patient 23# Song et al. (14)	2 years	Na	Seizure-free	NA
Case Li et al. (13)	6 months	Generalized tonic–clonic seizure	Seizure-free	Sporadic, low-amplitude slow spike-and-wave discharges in the central parietal and midline regions during sleep
Case Herzog et al. (8)	Birth	Myoclonus epilepsy, absence seizures	Seizure-free	Occipital hypersynchronous activity bilaterally with intermittent generalization without clinical correlates during the awake state and while asleep

*EEG, electroencephalogram; GTCS, generalized tonic–clonic seizures; NA, not available.*

However, considering the doubtful pathogenicity of c.1483G>T, we have not included the case reported by Xiaozhen et al. (16) (Patient 23) in our discussion of the clinical symptoms of all patients below. All patients with truncation mutations presented with epilepsy and varying degrees of delayed development. The age of epilepsy onset ranged from 6 months to 10 years, and various types of epilepsy were identified. Among the nine cases with treatment and prognosis data available, two cases were previously reported seizure-free after treatment (2/9, 22.2%), two cases were responsive to anti-seizure drugs (2/9, 22.2%), and five cases had refractory seizures (5/9, 55.6%). Interestingly, the case reported by Li et al. (13) was considered to be a case of febrile seizure; in this patient, a generalized tonic–clonic seizure occurred only once, and the patient was seizure-free without any anti-seizure medication (ASM). Conversely, many other patients developed seizures at different ages and did not respond well to drugs. Furthermore, most other patients had several types of seizures and did not respond to medication. Notably, myoclonus, and atypical absence occurred both alternately and simultaneously in our case.

At present, PME treatment remains palliative, supportive, and rehabilitative, with the best current therapies having limited success in the management of symptoms. Valproic acid is the most used ASM; it has a broad range of antiepileptic activities and is effective in treating myoclonus in various epileptic syndromes. Therefore, it is usually effective in suppressing, at least temporarily, most generalized tonic–clonic seizures as well as myoclonus to some extent. The other ASMs include lamotrigine, phenobarbital, and primidone, which at high doses can cause a level of cognitive impairment that is worse than the original condition for which they were being administered, and levetiracetam (3). Clonazepam is useful but often leads to considerable sedation and increasing tolerance. Due to the partial response to clonazepam and the short-term improvement after treatment with valproic acid and levetiracetam, myoclonus tends to persist after treatment (8). Some newer ASMs are reportedly effective, such as piracetam (17), topiramate (18), zonisamide (19), clobazam (20), and perampanel (21). Some ASMs can induce or worsen myoclonus (22), and these drugs should be avoided, particularly sodium-channel blockers (phenytoin, carbamazepine, oxcarbazepine, and lamotrigine), certain GABAergic drugs (tiagabine and vigabatrin), and gabapentin and pregabalin (23–26). It remains unclear why these drugs exacerbate myoclonus.

NCSE is a major but easily overlooked problem in PME. It may be associated with brain damage and developmental regression, and the treatment of NCSE is particularly important as a part of PME treatment. The traditional drug therapies mainly include diazepam, phenobarbital, and midazolam; however, they had no effect in our case. Instead, high-dose piracetam quickly improved the child’s EEG and returned their motor and language capabilities to baseline levels. Therefore, we speculate that piracetam may be effective against NSCE in patients with PME; however, this needs to be supported by other case reports and/or clinical trials. Nevertheless, to our knowledge, this is the first report about the treatment of NCSE in *SEMA6B*-related PME.

In conclusion, this report adds to the list of pathogenic *SEMA6B* variants and provides a better understanding of how different genotypes are related to the etiology of PME. These findings have important implications for the clinical management of patients with PME.

## Data Availability Statement

The datasets for this article are not publicly available due to concerns regarding participant/patient anonymity. Requests to access the datasets should be directed to LC, chenli2000@126.com.

## Ethics Statement

The studies involving human participants were reviewed and approved by Ethics Committee of Shenzhen Children’s Hospital. Written informed consent from the participants’ legal guardian/next of kin was not required to participate in this study in accordance with the national legislation and the institutional requirements.

## Author Contributions

LC was the first clinician to meet the patient when she was transferred to the hospital. JD was in charge to interpret the genetic data and drafted the manuscript along with CLiu. XZ, WZ, and QZ participated in case collection. YY, ZH, YC, CLi, TZ, JM, YX, YS, and JL were members of the treatment team of this patient and participated in the revision of the manuscript. All authors have read and approved the final manuscript.

## Conflict of Interest

CLiu and WZ were employed by the Berry Genomics Co., Ltd. The remaining authors declare that the research was conducted in the absence of any commercial or financial relationships that could be construed as a potential conflict of interest.

## Publisher’s Note

All claims expressed in this article are solely those of the authors and do not necessarily represent those of their affiliated organizations, or those of the publisher, the editors and the reviewers. Any product that may be evaluated in this article, or claim that may be made by its manufacturer, is not guaranteed or endorsed by the publisher.

## Abbreviations

PME: progressive myoclonic epilepsy
ASM: anti-seizure medication
NCSE: non-convulsive status epilepticus
ACMG: American College of Medical Genetics.
